# Influx of diverse, drug resistant and transmissible *Plasmodium falciparum* into a malaria-free setting in Qatar

**DOI:** 10.1186/s12879-020-05111-6

**Published:** 2020-06-15

**Authors:** Abir Al-Rumhi, Zainab Al-Hashami, Salama Al-Hamidhi, Amal Gadalla, Raeece Naeem, Lisa Ranford-Cartwright, Arnab Pain, Ali A. Sultan, Hamza A. Babiker

**Affiliations:** 1 Department of Biochemistry, College of Medicine and Health Sciences, Sultan Qaboos University, Muscat, Oman; 2 Biological and Environmental Sciences and Engineering Division, King Abdulla University for Science and Technology (KAUST), Thuwal, Kingdom of Saudi Arabia; 3 Institute of Biodiversity, Animal Health and Comparative Medicine, College of Medical, Veterinary and Life Sciences, University of Glasgow, Glasgow, G12 8QQ Scotland, UK; 4 Research Centre for Zoonosis Control, Global Institution for Collaborative Research and Education (GI-CoRE), Hokkaido University, N20 W10 Kita-ku, Sapporo, Japan; 5 Nuffield Division of Clinical Laboratory Sciences (NDCLS), The John Radcliffe Hospital, University of Oxford, Headington, Oxford, OX3 9DU UK; 6 Department of Microbiology and Immunology, Weill Cornell Medicine – Qatar, Cornell University, Qatar Foundation - Education City, Doha, Qatar; 7 Institute of Immunology and Infection Research, School of Biological Sciences, University of Edinburgh, Edinburgh, UK

**Keywords:** Gulf cooperation council (GCC) countries, Imported malaria, Malaria elimination, *P. falciparum*, Gametocytes, Qatar

## Abstract

**Background:**

Successful control programs have impeded local malaria transmission in almost all Gulf Cooperation Council (GCC) countries: Qatar, Bahrain, Kuwait, Oman, the United Arab Emirates (UAE) and Saudi Arabia. Nevertheless, a prodigious influx of imported malaria via migrant workers sustains the threat of local transmission. Here we examine the origin of imported malaria in Qatar, assess genetic diversity and the prevalence of drug resistance genes in imported *Plasmodium falciparum,* and finally, address the potential for the reintroduction of local transmission.

**Methods:**

This study examined imported malaria cases reported in Qatar, between 2013 and 2016. We focused on *P. falciparum* infections and estimated both total parasite and gametocyte density, using qPCR and qRT-PCR, respectively. We also examined ten neutral microsatellites and four genes associated with drug resistance, *Pfmrp1*, *Pfcrt*, *Pfmdr1*, and *Pfkelch13,* to assess the genetic diversity of imported *P. falciparum* strains, and the potential for propagating drug resistance genotypes respectively.

**Results:**

The majority of imported malaria cases were *P. vivax*, while *P. falciparum* and mixed species infections (*P. falciparum* / *P. vivax*) were less frequent. The primary origin of *P. vivax* infection was the Indian subcontinent, while *P. falciparum* was mostly presented by African expatriates. Imported *P. falciparum* strains were highly diverse, carrying multiple genotypes, and infections also presented with early- and late-stage gametocytes. We observed a high prevalence of mutations implicated in drug resistance among these strains, including novel SNPs in *Pfkelch13*.

**Conclusions:**

The influx of genetically diverse *P. falciparum,* with multiple drug resistance markers and a high capacity for gametocyte production, represents a threat for the reestablishment of drug-resistant malaria into GCC countries. This scenario highlights the impact of mass international migration on the reintroduction of malaria to areas with absent or limited local transmission.

## Background

The Gulf Cooperation Council (GCC) countries have been successful in malaria control. The increased investments in control efforts beginning in the 1950s, are largely responsible for the interruption of local transmission, and ultimately led to malaria-free status in four of the six GCC countries. In Saudi Arabia, limited foci of indigenous malaria still exist [1, 2], and in Oman sporadic outbreaks still occur periodically [3]. These successes have encouraged health ministries in GCC countries to shift policy toward a malaria-free Arabian Peninsula [4] and to focus on preventing reintroduction via sustainable vector control policy, improved surveillance, and prompt case management [5].

Qatar has been free from local malaria transmission since the 1970s [6], with no reports of autochthonous malaria [7]. Despite this, the influx of migrant workers from malaria-endemic countries of the Indian subcontinent and sub-Saharan Africa has sustained a relatively high number of imported cases, representing a major threat for the restoration of local transmission patterns. Migrant workers constitute the majority of residents in most GCC countries, reaching > 80% of the populace in Qatar [8]. Over the past two decades there has been an increase in the flow of migrant workers to Qatar in particular. This migratory trend has been associated with a positive trend in reported cases of imported malaria [7, 9, 10]. In addition to an increase in imported malaria, the receptivity and risk of malaria reintroduction is evident by the continued presence of the mosquito vectors *Anopheles stephensi* and *An. multicolor* [11].

Similar risk factors exist in other GCC countries, with relatively high percentages of expatriates from malarious areas. Many migrant freelance labourers from the Indian subcontinent are subject to crowded, sub-standard living conditions, work in stressful jobs in construction and agriculture that undermine immune health, and live in close proximity to irrigation sites or water tanks, thus having an increased exposure to mosquito vectors [3]. In Oman, there have been malaria outbreaks, presumably seeded from imported cases, as a consequence of these conditions, with infected individuals not identified and treated early enough to prevent transmission to the indigenous *Anopheles* population [3].

The chronic nature of asymptomatic malaria infection, common in adults from malaria-endemic countries, in conjunction with population mobility, creates a threat to effective, long-term malaria elimination [12]. Imported asymptomatic infection often carries drug-resistant strains of the *Plasmodium* parasite [9, 13], posing an imminent threat to receptive regions where local transmission has been eliminated or targeted for elimination [14]. An improved understanding of transmission patterns, extending to parasite genetic diversity, and the prevalence of drug-resistant strains of imported malaria aids the deployment of effective cross-border mitigation measures.

The present study examines the source of imported malaria to the transmission-free country of Qatar, and assesses the genetic diversity, prevalence of drug resistance mutations, and ability of *P. falciparum* to produce gametocytes and thus be transmitted to mosquito vectors. Such knowledge would allow control programs to develop targeted policies to reduce circulating parasites, define the source of outbreaks and limit the risk of reintroduction of malaria.

## Methods

### Subjects

A total of 583 patients reporting to either Hamad General Hospital or Al-Khor Hospital, two main centers of Hamad Medical Corporation (HMC), Doha, Qatar, were tested for malaria between January 2013 and October 2016. All malaria cases were diagnosed using microscopic examination of Giemsa-stained thick (100 fields) and thin blood (1000 RBC) films. A total of 448 (76.8%) of subjects tested positive for malaria, and based on disease history questionnaires, the origin of infection in all patients originated in endemic regions outside of Qatar [15]. Genomic DNA from capillary blood was isolated and purified using a QIAamp DNA bloodmini kit, following the manufacturer’s instructions (Qiagen, CA, USA). *Plasmodium* species identification was confirmed using species-specific PCR, as described elsewhere [16]. Quantitation of *P. falciparum* was carried out by qPCR of *18 s rRNA* [17]. Demographic information on each subject was ascertained using a survey, including variables such as age, sex, nationality, travel history, and previous malaria diagnoses/treatments. Patients who tested positive were provided with appropriate antimalarial treatment, according to the current, standard guidelines at HMC [11].

### Microsatellite genotyping and multiplicity of infection (MOI)

A panel of ten unlinked polymorphic microsatellites of *P. falciparum* were genotyped as described by Anderson and colleagues [17, 18]. PCR products were subjected to capillary electrophoresis using an ABI 3130XL Genetic Analyzer (Applied Biosystems, UK). Gene Mapper software version 4 (Applied Biosystems, UK) was used to score allele size and quantify electropherogram peak heights for samples containing multiple alleles per locus. Multiple alleles per locus were scored if an electrophoretic peak corresponding to a minor allele was > 32% of the height of the predominant allele [17].

### Detection and quantitation of early- and late-stage *P. falciparum* gametocytes

Quantitative RT-PCR (qRT-PCR) was employed to detect and quantify mRNA from the early gametocyte-specific gene, *Pfpeg4* [19], and the late gametocyte-specific gene, *pfs25* [20]. Total RNA was first isolated from 100 μL of capillary blood (collected via finger stick) using an SV Total RNA Isolation System (Promega, U.K.). Quantitative reverse transcription and subsequent amplification (qRT-PCR) of cDNA was carried out using a High Capacity cDNA Reverse Transcription Kit (Thermo Fisher, U.K.). RT-PCR conditions and primers were those previously described by Hemson et al. and Schneider et al. [21, 22].

### Amplicon sequencing for the characterization of *P. falciparum* drug resistance loci

SNPs in four *P. falciparum* genes implicated in resistance to several antimalarial drugs, *Pfmrp1* (PF3D7_0112200), *Pfcrt* (PF3D7_0709000), *Pfmdr1* (PF3D7_0523000), and *PfK13* (PF3D7_1343700), were typed according to the methodology of Rao et al. [23]. Multiplex PCR was combined with custom-designed sequence analysis using the Illumina® Miseq sequencing protocol (for the high-throughput SNP profiling of drug resistance genes) [23]. Seventy *P. falciparum* isolates were examined together with two controls, laboratory *P. falciparum* clones 3D7 and Dd2, with known alleles of the examined genes and phenotypic responses to antimalarials [23]. The *PfK13* and *Pfcrt* genes were each amplified as single fragments, while the longer *Pfmdr1* and *Pfmrp1*genes were each amplified as two fragments. PCR was carried out using the following conditions, in a total volume of 25 μl: 1 μl (10 pmol) of primers, 0.4 μl of dNTPs (200 μmol/L), 4 μl of Phusion HF buffer (5x) and 1 U of Phusion high-fidelity polymerase enzyme. The cycling profile for all loci was as follows: 98 °C / 30 s, followed by 30 cycles of (98 °C / 10 s, 64 °C / 4 min.), and a final extension of 64 °C / 5 min. The PCR amplicons of all genes, for each isolate, were pooled and purified using an Agencourt AMPure XP purification system and quantified using a Qubit double-stranded DNA (dsDNA) HS assay kit (Thermo Fisher Scientific). Sequencing was conducted on Illumina® MiSeq systems. Initially, libraries were prepared using a Nextera XT kit, according to the manufacturer’s protocol (Illumina Nextera® XT DNA Sample Preparation Guide, 2012). Following PCR cleanup, libraries were quantified using a Qubit dsDNA BR kit, and evaluated for fragment size using an Agilent High Sensitivity DNA Kit, designed for the 2100 Bioanalyzer (Agilent Technologies, Santa Clara, CA, USA). Each library was normalized for sequencing to 10 pM, according to the manufacturer’s protocol, and following Illumina’s (Illumina®, SanDiego, CA, USA) directions for cluster optimization (Illumina Nextera® Library Validation and Cluster Density Optimization, 2013). Sequencing reactions were carried out using a MiSeqReagent Kit V2, for 50 cycles (MiSeq, Illumina). SNPs in all genes were called using the reference sequence of the *P. falciparum* 3D7 clone, version 3 (PlasmoDB, PF3D7 v3).

### Data analysis

All samples that contained gametocyte transcripts, as detected by qRT-PCR, were assessed for association between gametocyte carriage and total parasitemia. A Mann-Whitney U test was used to examine the difference in density between early- and late-stage gametocytes. Spearman’s rank correlation was used to test for association between total parasite density (*18S rRNA* copy number) and the density of both late-stage gametocytes (*Pfs25* copy number) and early-stage gametocytes (*Pfpeg4* copy number). Microsatellite data was filtered to retain only minor alleles having a peak height of > 33% of the predominant allele, if more than one allele was present at a given locus. Genetic diversity metrics were primarily calculated using GenAlEx v6.5 [24]. Expected heterozygosity was calculated using the formula for ‘unbiased heterozygosity’ also termed haploid genetic diversity, *He* = [*n*/(*n*-1)][1-*Σp*^2^], where *n* is the number of isolates and *p* is the frequency of each allele at a given locus [25]. Population differentiation was assessed using Wright’s *FST* index in *F*stat version 2.9.3.2. Two estimators of *FST* (*G′ST* and *θ*) [26, 27] were used to estimate genetic differentiation between imported parasites from the Indian subcontinent and imported parasites from sub-Saharan Africa. Multiplicity of infection (MOI), defined as the presence of multiple genotypes per infection, was assessed through the detection of multiple alleles at a given locus. To avoid the over estimation of low-abundance alleles, only minor alleles having a peak height of > 33% of the corresponding predominant alleles were accepted. The proportion of samples with more than one allele across ten loci was used to represent MOI. The maximum number of alleles across the ten loci was used as an index for minimum number of clones per infection (MNC). The overall mean of the index value for each sample was then calculated.

## Results

### Demographic characteristics of imported malaria cases in Qatar

Among the 583 patients (all expatriates) tested for malaria between January 2013 and October 2016 in Hamad General Hospital and Al-Khor Hospital, Doha, Qatar, 448 (76.8%) tested positive for malaria: 318 for *P. vivax* (70.9%), 118 for *P. falciparum* (26.3%) and 12 (2.7%) for *P. vivax* / *P. falciparum* coinfection (Table 1) (Supplementary Table 1).

**Table 1:** Origin of imported malaria cases in Qatar between 2013 and 2016. The percentage values in brackets represent the proportion of one species originating from the country listed. The information on the originating country of the expatriates was obtained in response to the questionnaire and may not reflect all countries through which the individual travelled prior to arrival in Qatar

Country	Origin	***P. vivax (%)***	***P. falciparum (%)***	***P. vivax + P. falciparum***
The Indian Subcontinent	India	148 (46.5%)	18 (15.3%)	2
	Pakistan	104 (32.7%)	4 (3.4%)	0
	Sri Lanka	0	1 (0.8%)	0
	Nepal	12 (3.8%)	1 (0.8%)	0
Africa	Mauritania	1 (0.3%)	0	0
	Sudan	34 (10.7%)	36 (30.5%)	4
	Kenya	3 (0.9%)	16 (13.6%)	3
	Nigeria	3 (0.9%)	11 (9.3%)	2
	Eritrea	5 (1.6%)	10 (8.5%)	1
	Ethiopia	5 (1.6%)	3 (2.5%)	0
	Ghana	1 (0.3%)	3 (2.5%)	0
	Rwanda	0	2 (1.7%)	0
	Cameroon	0	2 (1.7%)	0
	Tanzania	1 (0.3%)	1 (0.8%)	0
	Djibouti	0	1 (0.8%)	0
	Democratic Republic of Congo	0	1 (0.8%)	0
	Republic of Ivory Coast	0	1 (0.8%)	0
	Chad	0	1 (0.8%)	0
Others^a^	Romania	0	1 (0.8%)	0
	USA	0	1 (0.8%)	0
	Syria	0	1 (0.8%)	0
	Qatar	0	1 (0.8%)	0
	Saudi Arabia	0	1 (0.8%)	0
	Spain	0	1(0.8%)	0
	Canada	1(0.3%)	0	0
Total		318	118	12

Others^a^: Reported by patients who have been on a visit to malaria-endemic countries

The primary origin of those presenting with *P. vivax* was the Indian subcontinent: India (46.0%, *n* = 146), Pakistan (32.1%, *n* = 102) and Nepal (3.8%, *n* = 12). A smaller proportion of *P. vivax* cases were from sub-Saharan Africa (16%, *n* = 53) (Table 1). Unlike *P. vivax*, the primary origin of *P. falciparum* infection was Africa: East Africa (76.1%, *n* = 67), West and Central Africa (23.9%, *n* = 21), followed by the Indian subcontinent (20.3%, *n* = 24) and other regions (5.1%, *n* = 6) (Table 1).

### Parasitaemia and gametocytaemia among imported malaria cases

Ninety of the 118 *P. falciparum* infections were examined for (1) total parasite density (qPCR), (2) total gametocyte density (qRT-PCR), (3) diversity within 10 microsatellites, and (4) four genes linked to drug resistance. The total *P. falciparum* density among imported cases varied widely, ranging between 32 and 9,218,498 parasites/ml of blood, with a median of 82,783 parasites/ml. The median parasite density among imported cases from the Indian subcontinent (99,572 parasites/ml) was not significantly different from the parasite density among imported cases from Africa (88,504 parasites/ml) (*P* = 0.394).

**Fig. 1:**
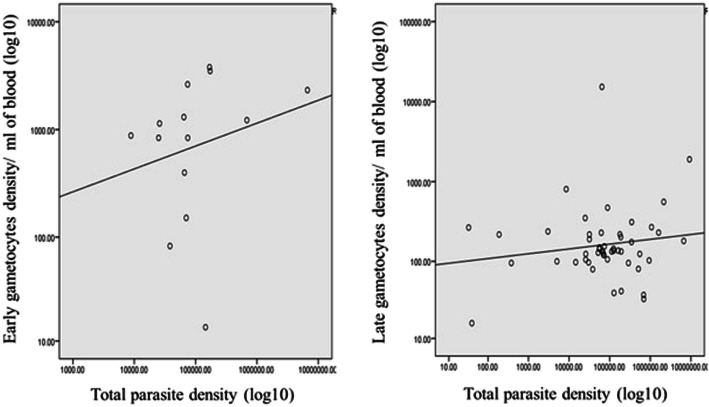
Correlation between total parasite density with both early gametocyte and late gametocyte density. **a** log total parasitaemia (X axis) and log early gametocyte density (Y axis), the fit line in scatter plot shows a weak/non-significant correlation coefficient (r = 0.031, *p =* 0.835). **b** log total parasitaemia (X axis) and log late gametocyte density (Y axis), the fit line in scatter plot shows a weak/non-significant correlation coefficient (r = 0.008, *p* = 0.946)

Seventy-three *P. falciparum* isolates were successfully examined by qRT-PCR to detect and quantify transcripts of genes expressed in early- (*Pfpeg4*) and late-stage gametocytes (*Pfs25*). The prevalence of all gametocytes was 74% (*n* = 54), with 9.6% (*n* = 7) of subjects possessing only early-stage gametocytes, 37% (*n* = 27) possessing only late-stage gametocytes, and 27.4% (*n* = 20) possessing a mixture of both stages. Early- stage gametocytes were found at a density ranging between 14 and 3781/ml blood, with a median of 1011/ml. Late-stage gametocytes were found at a density of ranging between 16 and 15,289/ml blood, with a median of 136 /ml blood). There was a significant difference in the density of early- and late-stage gametocyte densities (Mann-Whitney U test, *P* = 0.003). There was no correlation between total parasitaemia (18S rRNA copy number) and either late gametocyte density (*Pfs25* copy number) (r_s_ = 0.008, *P* = 0.946) or early gametocyte density (*Pfpeg4* copy number) (r_S_ = 0.031, *P* = 0.835) (Fig. 1).

### Genetic diversity and the structure of imported *P. falciparum*

#### Microsatellite polymorphism

All 10 microsatellites examined were highly polymorphic for *P. falciparum* isolates originating in sub-Saharan Africa and the Indian subcontinent (Table 2). The number of alleles per locus was greater among African isolates, ranging from 5 (*Pfg377*) to 18 (*polyα*), compared to isolates from the Indian subcontinent, that ranged from 3 (*2490*) to 7 (*TA1* and *PfPK2)* (Table 2; Supplementary Table 2). Nevertheless, allelic diversity (defined as the mean expected heterozygosity (*He*) across 10 microsatellite loci) was not significantly different among parasites from the Indian subcontinent (mean *He* = 0.78) compared to those from sub-Saharan Africa (mean *He* = 0.76) (*P* = 0.333).

**Table 2:** Number of alleles and expected heterozygosity (*He*) at ten microsatellite loci within imported *Plasmodium falciparum* from the Indian Subcontinent and Africa

Origin of isolates		*2490*	*Pfg377*	*polyα*	*TA109*	*TA81*	*ARA2*	*PfPK2*	*TA1*	*TA60*	*TA87*
The Indian Subcontinent (*n* = 13)	Alleles	3	4	6	5	5	5	7	7	6	6
	*He*	0.67	0.68	0.87	0.81	0.74	0.71	0.91	0.87	0.72	0.86
Africa (*n* = 77)	Alleles	7	5	18	10	10	11	9	13	6	10
	*He*	0.48	0.58	0.93	0.81	0.76	0.81	0.78	0.85	0..76	0.83

Multi-locus haplotypes were constructed using predominant alleles at all examined loci. All 90 isolates differed from each other by at least one examined locus, with the exception of two isolates from Sudan.

#### Multiplicity of infection (MOI)

Seventy-six (84.4%) of the 90 imported *P. falciparum* isolates with complete available data possessed multiple genotypes. The minimum number of genotypes per infected person (the mean maximum number of alleles observed at all loci) was slightly lower in sub-Saharan Africa (2.16 genotypes) than the Indian subcontinent (2.38 genotypes) (α < 0.05) (*P* = 0.667).

#### Genetic differentiation

**Fig. 2:**
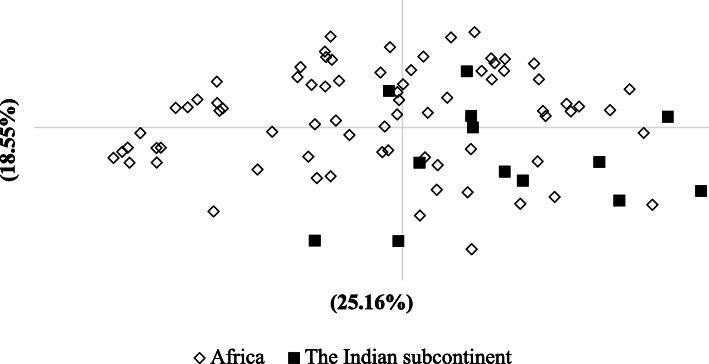
Principal Co-ordinates Analysis (PCoA) of *P. falciparum* populations from two regions (the Indian Subcontinent [black square] and Africa [transparent rectangular]). Values within parenthesis after the coordinate number are the percentage of variation explained by the coordinate

Alleles of most *P. falciparum* microsatellites were distributed widely among imported malaria cases from both sub-Saharan Africa (*n* = 77) and the Indian subcontinent (*n* = 13). A relatively large number of private alleles (alleles detected only in one geographic region) were seen in Africa (*n* = 50) compared to the Indian subcontinent (*n* = 5). This may reflect the smaller sample size of subjects with origins in the Indian continent. Despite this trend, no evidence of genetic differentiation was observed between imported *P. falciparum* from sub-Saharan Africa and that imported from the Indian subcontinent (*F*_*ST*_ = 0.055). The genetic relatedness between *P. falciparum* populations was assessed using PCoA analysis (Fig. 2), which showed overlap between two populations. Analysis of molecular variance (AMOVA) within *P. falciparum* isolates imported from the Indian subcontinent and Africa revealed that the majority of genetic variation occurred between individuals within populations (95%), compared to differences between populations (*P* < 0.001).

#### Distribution of drug resistance mutations

Seventy imported *P. falciparum* isolates were examined by amplicon sequencing for four putative drug resistance genes, *PfK13, Pfmdr1, Pfcrt and Pfmrp1* (Table 3). With the exception of *PfK13*, there was no difference in the prevalence of wild-type alleles among parasites originating from sub-Saharan Africa versus the Indian subcontinent. There was, however, a significantly higher prevalence of mutant *PfK13* haplotypes among parasites from Africa than the Indian subcontinent (*P* = 0.0036). One nonsynonymous mutation in *PfK13* (K189**T**) was observed at a prevalence of 36% among parasites originating from Sudan (*n* = 36), similar to reported findings from other African countries [28]. Ten additional nonsynonymous SNPs within *PfK13* were identified at prevalences ranging from 1 to 3%: K108**E** (2%), L119**L** (1%), H136**N** (1%), T149**S** (2%), K189**N** (2%), N217**H** (1%), R255**K** (3%), I354**V** (1%), E433**D** (1%) and G453**A** (1%) (Table 3; Supplementary Table 3).

**Table 3:** Haplotypes of drug resistance genes, that exist at a prevalence of more than 5%, among imported *P. falciparum* cases in Qatar. Haplotypes are shown as amino acids (wild-type in normal case, substitutions in bold underlined)

Locus	Genotype	**Haplotype**	Prevalence	the Indian subcontinent (*n* = 7)	Africa (*n* = 63)	*P* value
*Pfcrt*	Wild type	C_72_ K_76_ A_220_ Q_271_ N_326_ I_356_ R_371_	44%	1(14%)	30(48%)	0.1233
	Mutant	C_72_ K_76_**S**_220_**E**_271_ N_326_ I_356_ R_371_	13%	6(85%)	33(52%)	
	Mutant	C/S_72_**T**_76_**S**_220_**E**_271_ N_326_ I_356_ R_371_	5%			
	Mutant	C_72_ K_76_**S**_220_**E**_271_**S**_326_ I_356_ R_371_	29%			
*Pfmdr1*	Wild type	N_86_ F_184_ F_938_ G_968_ D_1246_	39%	4(57%)	23(37%)	0.4118
	Mutant	N_86_**Y**_184_ F_938_G_968_ D_1246_	10%	3(43%)	43(68%)	
	Mutant	**Y**_86_ F_184_ F_938_ G_968_ D_1246_	30%			
*PfK13*	Wild type	H_136_ T_149_ K_189_ N_217 R255_ E_433_G_453_	47%	7(100%)	26(41%)	0.0036
	Mutant	H_136 T149_**T**_**189**_ N_217_ R_255_ E_433_ G_453_	34%	0(0%)	37(59%)	
*Pfmrp1*	Wild type	H_191_K_202_S_437_I_876_L_1342_F_1390_ K_1466_D_1533_	31%	1(14%)	21(33%)	0.4201
	Mutant	H_191_K_202_S_437_**V**_876_L_1342_F_1390_ K_1466_D_1533_	6%	6(86%)	42(67%)	
	Mutant	H_191_K_202_S_437_**V**_876_L_1342_F_1390_**R**_1466_D_1533_	10%			
	Mutant	**Y**_191_K_202_**A**_437_**V**_876_L_1342_**I**_1390_ K_1466_D_1533_	5%			

*PfK13* variants including the substitutions C580**Y**, Y493**H**, R539**T** and M579**I**, previously shown to be associated with slow artemisinin clearance of *P. falciparum* [29, 30], were not detected among *P. falciparum* isolates in this study. However, mutations in the gene *Pfmdr1*, also associated with reduced ACT susceptibility in some studies [31], as well as in resistance to other antimalarial drugs such as chloroquine [32], were found in the imported parasites. The *Pfmdr1* polymorphisms N86**Y** and Y184**F** were prevalent among imported *P. falciparum* isolates (33 and 77%, respectively). In addition, six rare nonsynonymous SNPs were detected (see Table 3). The N_86_**F**_184_D_1246_ and **Y**_**8**6_**F**_184_D_1246_ haplotypes, associated with artemether-lumefantrine (AL) tolerance [33] and chloroquine/amodiaquine (CQ/AQ) treatment failure, were reported among imported *P. falciparum* cases, at 43 and 33%, respectively.

Notably, while the *Pfcrt* K76**T** substitution associated with CQR was found at a relatively low frequency (*n* = 70, 6%), other SNPs implicated in CQR were observed at higher prevalence: A220**S** (53%), Q271**E** (49%), N326**D**/**S** (36%), I356**L** (6%) and R371**I** (47%). Overall, the CQ sensitive haplotype C_72_V_73_M_74_N_75_K_76_ was common (94%), while the CQ resistant haplotypes, **S**_72_V_73_M_74_N_75_**T**_76_ and C_72_V_73_M_74_N_75_**T**_76_, were detected in only one and three isolates, respectively.

Regarding *Pfmrp1*, eight variants were observed among imported *P. falciparum* specimens, ranging from a frequency of 46% for I876**V** to 3% for D1533**V** (Table 3, Supplementary Table 3). The five most common variants [H191**Y**, S437**A**, I876**V**, F1390**I**, K1466**R**] detected among imported cases were all previously reported in the Indian subcontinent and Africa [34, 35], however, they existed at a relatively higher frequency in isolates from Africa compared to those from the Indian subcontinent (Supplementary Table 3). *Pfmrp1* polymorphisms previously associated with decreased in vitro susceptibility to SP, artemisinin, mefloquine, and lumefantrine were common. The most frequent SNP, encoding I876**V** (46%), was found to be under significant selection pressure following AL treatment [34].

## Discussion

Sustainable interventions driven by global support have resulted in a noteworthy global malaria case reduction in the past three decades [5]. Twenty-one countries were identified by the WHO for the complete elimination of malaria by the year 2020 [36]. Drug resistance in *Plasmodium* species and insecticide resistance in malaria vectors, as well as human sociodemographic factors, have the potential to obstruct this worthy goal. Mass international human migration from malaria-endemic regions where a proportion of semi-immune residents sustain asymptomatic, low levels of parasitemia has been shown to be a risk factor for the reintroduction of local malaria transmission.

Asymptomatic *P. falciparum* infection can develop into clinical malaria in individuals up to 8 years after migration to a malaria-free country [37, 38]. Therefore, asymptomatic migrants with malaria parasite infections can act as a long-lasting reservoir for secondary local transmission in receptive malaria-free areas, where elimination has been accomplished [3, 39]. This potential is evident in the high prevalence of gametocyte carriage seen among imported *P. falciparum* malaria cases in Qatar. Fifty-four of 73 imported *P. falciparum* isolates (74%) examined by qRT-PCR [40] possessed gametocyte stages, with a large proportion (37%) harboring both early-stage and late-stage gametocytes. This trend is indicative of ongoing gametocytogenesis from the asexual population present in the patient. Low-density gametocytes can readily infect *Anopheles* vectors, even at submicroscopic levels [41, 42]. Secondary transmission, arising from imported malaria cases, is often reported in GCC countries in areas where the *Anopheles* vector is present, and a favorable ecological habitat exists [3, 43]. The surge in *Anopheles* abundance in highly-seasonal transmission settings has been associated with an upsurge in gametocyte numbers in asymptomatic carriers [20]. This is in line with enhanced parasite infectivity in response to increased exposure to uninfected mosquitoes at the start of the transmission season, in areas with marked seasonal malaria [20, 44]. Although the resumption of endemic malaria transmission in GCC countries is unlikely given current models, the high rate of imported malaria can readily seed outbreaks, if vector control wanes [3].

The genetic diversity, measured as *H*_*e*,_ of *P. falciparum* imported into Qatar from sub-Saharan Africa (0.76) and the Indian subcontinent (0.78), is similar to that reported locally in both sites of origin [17, 40], as well as within local transmission sites that still exist in Saudi Arabia (0.76) and Yemen (0.585) [45]. This pattern is also found in genotype multiplicity. These trends underscore the role of imported malaria in enriching *Plasmodium* genetic diversity, as well as in introducing drug resistance lineages in areas moving towards elimination, such as Saudi Arabia [46] and Oman [3]. Moreover, the combination of high genotype multiplicity and gametocyte carriage, as reported in the present study, increases the likelihood that imported malaria infections will generate novel genotypes, should transmission occur [47]. Thus, imported malaria cases to Qatar represent not only a risk for the ignition of local transmission, but also a risk of creating novel strains that can escape the effect of current drug regimens. Efforts to thwart the reintroduction of malaria in transmission-free areas of the GCC currently rely on effective case management using artemisinin-combination therapy. The present study revealed a relatively high prevalence of SNPs in four unlinked genes implicated in drug resistance, namely *Pfcrt*, *Pfmdr1*, *Pfmrp1* and *PfK13*.

With the exception of *PfK13*, there was no difference in the distribution of drug resistance alleles between *Plasmodium* parasites introduced from the Indian subcontinent as compared to those from sub-Saharan Africa. The wild type allele of *PfK13* was found at high prevalence in *P. falciparum* imported from sub-Saharan Africa and the Indian subcontinent, and the variants C580**Y**, Y493**H**, R539**T**, and M579**I**, previously associated with slow artemisinin clearance [28, 30], were not detected.. Numerous low-frequency (1 to 3%) SNPs in *PfK13* in parasites from sub-Saharan Africa and the Indian subcontinent were observed(K108**E**, L119**L**, H136**N**, T149**S**, K189**N**, N217**H**, R255**K**, I354**V**, E433**D**, G453**A**), while one SNP, K189**T**, was observed at a conspicuously higher frequency (36%) among parasites originating specifically from Sudan [28]. However, parasites carrying mutation K189**T** have previously been found to have a similar therapeutic response (parasite clearance half-life) to ACT to wild type parasites [28], and thus may not have an impact on the current ACT regimen in Qatar for uncomplicated and complicated *P. falciparum* infection [11].

The presence of SNPs or haplotypes linked to tolerance of artemisinin derivatives can however impact on current elimination strategies, potentially resulting in persistence of symptoms and an increased local parasite reservoir. We reported a high prevalence of the *Pfmdr1*- N_86_**F**_184_D_1246_ haplotype (43%), which is associated with reduced AL susceptibility [31]. The two most common variants of *Pfmrp1*, F1390**I** (79%) and I876**V** (46%), have been linked to a decreased susceptibility to artemisinin, mefloquine, and lumefantrine [36, 48]. These findings suggest that regular monitoring of the above SNPs, coupled with an appropriate, tailored clinical response, should be employed to combat the spread of *P. falciparum* parasites with potential AL tolerance. Studies in Saudi Arabia and Yemen have revealed a high frequency of drug resistance genotypes among locally-acquired *P. falciparum* infections, the source of which was linked statistically to sub-Saharan Africa and Indian subcontinent [34, 48].

A noteworthy observation from the present study was the divergence in the frequency of *Pfcrt* mutation K76**T** (11%) compared to mutations A220**S** (54%), Q271**E** (50%), N326**D**/**S** (37%), and R371**I** (48%). Like K76**T**, the latter mutations have been associated with reduction in CQ transport activity and CQR [49]. The relatively low frequency of the K76**T** mutation may reflect the fitness cost of the variant, coupled with reduced exposure to CQ [50], while the other mutations may carry lower fitness costs and so be maintained in the population. Alternatively, the latter *Pfcrt* mutations may be under selective pressures from both CQ and other antimalarial drugs.

## Conclusions

The present study expounds upon the threat that imported *Plasmodium* parasites represent for the reintroduction of malaria in receptive, transmission-free areas, such as Qatar. The high levels of genetic diversity found, as well as the high capacity of imported *P. falciparum* to produce gametocytes, highlights the threat of spread of drug resistance genotypes, should local transmission reoccur. There is an urgent need for molecular tools, such as the highly sensitive and high-throughput qPCR that can detect > 20 parasites/ml blood [51], for the surveillance of imported malaria cases in Qatar and the GCC, to limit the risk of reintroduction of malaria.

## Supplementary information

**Additional file 1: Table S1.** Demographic data of imported *P. falciparum* malaria cases.

**Additional file 2: Table S2.** Allele size of 10 microsatellites among imported *P. falciparum* to Qatar.

**Additional file 3: Table S3.** Prevalence of wild-type and mutant alleles of drug resistance genes among imported *P. falciparum* to Qatar. Values in brackets are percentages.

## Data Availability

The datasets used in the current study are available from the corresponding author on reasonable request.

## Abbreviations

GCC: Gulf Cooperation Council
HMC: Hamad Medical Corporation
MOI: Multiplicity of infection
qRT-PCR: Quantitative Reverse Transcriptase-Polymerase Chain Reaction
MNC: Minimum number of clones per infection
H_*e*_: Expected heterozygosity
F_*ST*_: Fixation index
PCoA: Principle coordinates analysis
AMOVA: Analysis of molecular variance
ACT: Artemisinin-based combination therapy
AL: Artemether-Lumefantrine
CQ: Chloroquine
AQ: Amodiaquine
CQR: Chloroquine Resistance
SP: Sulfadoxine pyrimethamine
WHO: World Health Organization
