# Supplementary material for: Influx of diverse, drug resistant and transmissible Plasmodium falciparum into a malaria-free setting in Qatar
Source: BMC Infect Dis. 2020 Jun 15;20:413. doi: 10.1186/s12879-020-05111-6 (PMC7296620; doi:10.1186/s12879-020-05111-6)
Supplement: Supplementary file 1 — Additional file 1: Table S1. Demographic data of imported P. falciparum malaria cases. [file 12879_2020_5111_MOESM1_ESM.docx]

Supplementary Table 1: Demographic data of imported *P. falciparum* malaria cases

| **ID** | **Nationality** | **Collection center *** | **Treatment** | **Parasitemia** |
| --- | --- | --- | --- | --- |
|  |  |  |  | **(%)** |
| 1 | Indian | Al-Khor | Quinine plus Doxycycline | 0.7 |
| 2 | Eritrean | Al-Khor | Quinine plus Doxycycline | 0.6 |
| 3 | Eritrean | HMC | Coartem and doxycycline | 3.4 |
| 4 | Sudanese | HMC | Chloroquine | 2.1 |
| 5 | Kenyan | HMC | No available records | No available records |
| 6 | Sudanese | HMC | No available records | No available records |
| 7 | Ghanaian | HMC | Quinine and Coartem | 0.2 |
| 8 | Kenyan | HMC | Quinine plus Doxycycline | 4.9 |
| 9 | Kenyan | HMC | Quinine plus Doxycycline | 0.3 |
| 10 | Spanish | HMC | Coartem | 3.2 |
| 11 | Cameroonian | HMC | Coartem | 4.2 |
| 12 | Romanian | HMC | Quinine plus Doxycycline | 21.6 |
| 13 | Nigerian | HMC | Coartem | 0.1 |
| 14 | Nigerian | HMC | Coartem | 0.9 |
| 15 | Nigerian | HMC | Quinine | 2.3 |
| 16 | Sudanese | HMC | Quinine plus Clindamycin | 0.1 |
| 17 | Sudanese | HMC | Coartem plus Clindamycin | 0.6 |
| 18 | Sudanese | HMC | Quinine | 2.5 |
| 19 | Sudanese | HMC | Coartem | 1.2 |
| 20 | Nigerian | HMC | Quinine plus Doxycycline | 1.1 |
| 21 | Sudanese | HMC | No available records | 4.7 |
| 22 | Indian | HMC | Quinine plus Coartem | 0.6 |
| 23 | Sudanese | HMC | No available records | 1.2 |
| 24 | Kenyan | HMC | Quinine | 0.3 |
| 25 | Sudanese | HMC | Coartem | 1.1 |
| 26 | Sudanese | HMC | Quinine | 8.8 |
| 27 | Sudanese | HMC | Coartem | 0.6 |
| 28 | Sudanese | HMC | Coartem | 0.3 |
| 29 | Sudanese | HMC | Chloroquine | 1.9 |
| 30 | Eritrean | HMC | Quinine plus Doxycycline | 0.6 |
| 31 | Eritrean | HMC | Coartem | 0.6 |
| 32 | Pakistani | HMC | Coartem plus Clindamycin | 0.4 |
| 33 | Sudanese | HMC | No available records | 0.3 |
| 34 | Indian | HMC | No available records | 1 |
| 35 | Sudanese | HMC | Coartem | 2.4 |
| 36 | Eritrean | HMC | Coartem | 0.6 |
| 37 | Sudanese | HMC | Chloroquine | 0.2 |
| 38 | Eritrean | HMC | Coartem | 1.7 |
| 39 | Sudanese | HMC | No available records | 4.1 |
| 40 | Sudanese | HMC | no treatment (pt refused) | 0.8 |
| 41 | Sudanese | HMC | Coartem | 0.4 |
| 42 | Sudanese | HMC | no record | 0.1 |
| 43 | Sudanese | HMC | Coartem | 19.5 |
| 44 | Eritrean | HMC | Coartem | 0.2 |
| 45 | Ivory Coast | HMC | Coartem | 1.8 |
| 46 | Kenyan | HMC | Coartem | 0.1 |
| 47 | Eritrean | HMC | Coartem | 0.6 |
| 48 | Rwandan | HMC | Coartem | 0.2 |
| 49 | Sudanese | HMC | Coartem | 0.2 |
| 50 | Sudanese | HMC | Coartem | 0.1 |
| 51 | Congolian | HMC | Artesunate | 0.1 |
| 52 | Nigerian | HMC | Coartem | 1.8 |
| 53 | Sudanese | HMC | Coartem plus Primaquine | 2.2 |
| 54 | Ethiopian | HMC | Coartem | 1.2 |
| 55 | Kenyan | HMC | Coartem | 2 |
| 56 | Ethiopian | HMC | Coartem | 0.3 |
| 57 | Kenyan | HMC | Coartem | 0.2 |
| 58 | Ghanian | HMC | No available records | 0.8 |
| 59 | Kenyan | HMC | Coartem | 1.7 |
| 60 | Ghanian | HMC | Chloroquine | 0.1 |
| 61 | Eritrean | HMC | No available records | 5 |
| 62 | Sudanese | HMC | No available records | 2.8 |
| 63 | Pakistani | HMC | No available records | 2.7 |
| 64 | American | Al-Khor | No available records | 0.6 |
| 65 | Kenyan | HMC | No available records | 0.4 |
| 66 | Indian | Al-Khor | No available records | 0.1 |
| 67 | Kenyan | HMC | No available records | 5.6 |
| 68 | Kenyan | HMC | No available records | 2.9 |
| 69 | Djiboutian | HMC | No available records | 6.1 |
| 70 | Indian | HMC | No available records | 2.2 |

*HMC = Hamad Medical Cooperation

Al-Khor hospital
