# Supplementary material for: Influx of diverse, drug resistant and transmissible Plasmodium falciparum into a malaria-free setting in Qatar
Source: BMC Infect Dis. 2020 Jun 15;20:413. doi: 10.1186/s12879-020-05111-6 (PMC7296620; doi:10.1186/s12879-020-05111-6)
Supplement: Supplementary file 2 — Additional file 2: Table S2. Allele size of 10 microsatellites among imported P. falciparum to Qatar. [file 12879_2020_5111_MOESM2_ESM.docx]

**Supplementary Table 2.** Allele size of 10 microsatellites among imported *P. falciparum* to Qatar.

| **Loci/ population** | ***2490*** | ***Pfg377*** | ***polyα*** | ***TA109*** | ***TA81*** | ***ARA2*** | ***PfPK2*** | ***TA1*** | ***TA60*** | ***TA87*** |
| --- | --- | --- | --- | --- | --- | --- | --- | --- | --- | --- |
| Africa | 83 | 95 | 176 | 164 | 124 | 67 | 170 | 166 | 74 | 0 |
| Africa | 83 | 98 | 176 | 161 | 115 | 76 | 167 | 166 | 80 | 101 |
| Africa | 92 | 98 | 152 | 161 | 121 | 64 | 170 | 160 | 80 | 98 |
| Africa | 83 | 98 | 134 | 176 | 124 | 58 | 167 | 169 | 71 | 101 |
| Africa | 83 | 98 | 152 | 173 | 118 | 64 | 161 | 169 | 80 | 107 |
| Africa | 86 | 95 | 170 | 176 | 139 | 67 | 161 | 166 | 71 | 101 |
| Africa | 83 | 98 | 158 | 161 | 127 | 58 | 161 | 163 | 71 | 107 |
| Africa | 83 | 95 | 170 | 161 | 124 | 64 | 167 | 169 | 86 | 98 |
| Africa | 83 | 95 | 176 | 164 | 124 | 0 | 170 | 166 | 74 | 107 |
| Africa | 83 | 95 | 173 | 161 | 124 | 85 | 161 | 172 | 71 | 101 |
| Africa | 86 | 98 | 164 | 176 | 124 | 79 | 164 | 166 | 71 | 98 |
| Africa | 83 | 98 | 176 | 173 | 136 | 73 | 170 | 196 | 71 | 92 |
| Africa | 80 | 98 | 131 | 173 | 124 | 58 | 170 | 172 | 74 | 107 |
| Africa | 83 | 98 | 179 | 173 | 124 | 67 | 161 | 154 | 71 | 107 |
| Africa | 77 | 101 | 143 | 176 | 121 | 67 | 164 | 172 | 74 | 98 |
| Africa | 83 | 98 | 152 | 161 | 121 | 61 | 185 | 178 | 71 | 113 |
| Africa | 83 | 95 | 152 | 173 | 115 | 67 | 185 | 169 | 71 | 104 |
| Africa | 83 | 95 | 173 | 164 | 124 | 67 | 161 | 166 | 71 | 101 |
| Africa | 83 | 98 | 164 | 161 | 118 | 85 | 161 | 166 | 83 | 104 |
| Africa | 83 | 95 | 179 | 164 | 124 | 64 | 170 | 166 | 74 | 107 |
| Africa | 83 | 95 | 176 | 164 | 124 | 64 | 170 | 172 | 74 | 107 |
| Africa | 80 | 98 | 137 | 173 | 121 | 70 | 158 | 178 | 80 | 107 |
| Africa | 86 | 95 | 170 | 176 | 124 | 67 | 161 | 169 | 71 | 101 |
| Africa | 83 | 95 | 155 | 161 | 115 | 67 | 161 | 166 | 74 | 107 |
| Africa | 83 | 98 | 164 | 185 | 133 | 58 | 164 | 166 | 71 | 101 |
| Africa | 83 | 95 | 155 | 173 | 124 | 64 | 170 | 166 | 83 | 101 |
| Africa | 83 | 95 | 167 | 176 | 127 | 73 | 164 | 172 | 83 | 95 |
| Africa | 83 | 92 | 131 | 176 | 121 | 70 | 164 | 172 | 86 | 101 |
| Africa | 83 | 98 | 164 | 191 | 124 | 64 | 170 | 157 | 71 | 98 |
| Africa | 83 | 95 | 164 | 161 | 124 | 73 | 161 | 154 | 71 | 107 |
| Africa | 83 | 95 | 179 | 164 | 124 | 64 | 158 | 181 | 74 | 107 |
| Africa | 83 | 98 | 143 | 164 | 124 | 67 | 161 | 166 | 80 | 101 |
| Africa | 80 | 98 | 164 | 173 | 115 | 64 | 158 | 166 | 80 | 104 |
| Africa | 83 | 98 | 173 | 176 | 124 | 67 | 161 | 154 | 71 | 101 |
| Africa | 83 | 95 | 134 | 176 | 124 | 64 | 161 | 172 | 74 | 107 |
| Africa | 83 | 95 | 152 | 176 | 127 | 79 | 164 | 166 | 74 | 95 |
| Africa | 80 | 98 | 173 | 173 | 115 | 67 | 161 | 160 | 80 | 107 |
| Africa | 74 | 0 | 149 | 161 | 118 | 67 | 170 | 166 | 80 | 98 |
| Africa | 83 | 98 | 161 | 176 | 118 | 64 | 179 | 163 | 80 | 107 |
| Africa | 83 | 98 | 164 | 161 | 115 | 58 | 161 | 166 | 83 | 104 |
| Africa | 86 | 95 | 170 | 176 | 124 | 67 | 161 | 169 | 71 | 101 |
| Africa | 83 | 95 | 161 | 161 | 124 | 64 | 161 | 166 | 80 | 104 |
| Africa | 83 | 98 | 173 | 161 | 124 | 85 | 161 | 178 | 71 | 107 |
| Africa | 86 | 98 | 161 | 161 | 124 | 70 | 170 | 193 | 80 | 98 |
| Africa | 83 | 95 | 176 | 164 | 124 | 64 | 170 | 163 | 74 | 107 |
| Africa | 83 | 104 | 155 | 167 | 115 | 70 | 161 | 166 | 71 | 95 |
| Africa | 86 | 101 | 155 | 161 | 124 | 82 | 161 | 163 | 80 | 107 |
| Africa | 83 | 98 | 170 | 161 | 124 | 64 | 0 | 160 | 71 | 89 |
| Africa | 83 | 95 | 176 | 161 | 124 | 85 | 161 | 178 | 71 | 101 |
| Africa | 80 | 95 | 176 | 164 | 124 | 64 | 161 | 172 | 74 | 98 |
| Africa | 83 | 98 | 179 | 161 | 115 | 67 | 161 | 163 | 80 | 104 |
| Africa | 83 | 98 | 179 | 164 | 124 | 64 | 170 | 163 | 74 | 101 |
| Africa | 83 | 98 | 146 | 164 | 124 | 64 | 170 | 160 | 74 | 101 |
| Africa | 80 | 98 | 179 | 176 | 121 | 67 | 167 | 169 | 74 | 95 |
| Africa | 83 | 98 | 155 | 230 | 121 | 85 | 161 | 175 | 74 | 98 |
| Africa | 83 | 95 | 176 | 0 | 118 | 70 | 161 | 166 | 80 | 116 |
| Africa | 83 | 98 | 167 | 161 | 121 | 85 | 155 | 166 | 80 | 107 |
| Africa | 83 | 101 | 164 | 161 | 127 | 70 | 164 | 178 | 74 | 95 |
| Africa | 95 | 98 | 176 | 197 | 121 | 58 | 0 | 157 | 83 | 101 |
| Africa | 83 | 98 | 152 | 161 | 121 | 64 | 167 | 169 | 71 | 104 |
| Africa | 83 | 98 | 164 | 173 | 115 | 67 | 164 | 172 | 80 | 95 |
| Africa | 80 | 95 | 182 | 176 | 115 | 70 | 0 | 172 | 71 | 101 |
| Africa | 74 | 92 | 170 | 161 | 124 | 55 | 158 | 160 | 83 | 107 |
| Africa | 80 | 101 | 152 | 191 | 121 | 64 | 161 | 169 | 74 | 92 |
| Africa | 83 | 98 | 134 | 173 | 112 | 64 | 167 | 166 | 65 | 92 |
| Africa | 83 | 95 | 146 | 164 | 124 | 64 | 170 | 163 | 74 | 101 |
| Africa | 83 | 92 | 161 | 176 | 121 | 58 | 164 | 169 | 83 | 101 |
| Africa | 80 | 0 | 152 | 185 | 118 | 70 | 158 | 169 | 80 | 95 |
| Africa | 80 | 98 | 158 | 173 | 115 | 64 | 164 | 175 | 71 | 107 |
| Africa | 80 | 98 | 125 | 197 | 118 | 70 | 164 | 190 | 74 | 101 |
| Africa | 80 | 98 | 149 | 161 | 109 | 64 | 164 | 196 | 71 | 119 |
| Africa | 83 | 98 | 167 | 164 | 127 | 64 | 164 | 196 | 83 | 104 |
| Africa | 83 | 95 | 149 | 185 | 121 | 58 | 182 | 0 | 71 | 92 |
| Africa | 83 | 98 | 152 | 176 | 133 | 67 | 158 | 169 | 80 | 104 |
| Africa | 83 | 98 | 149 | 173 | 121 | 67 | 161 | 172 | 80 | 104 |
| Africa | 83 | 98 | 152 | 185 | 124 | 64 | 161 | 166 | 83 | 116 |
| Africa | 83 | 98 | 149 | 158 | 118 | 85 | 179 | 166 | 80 | 92 |
| The Indian subcontinent | 80 | 92 | 152 | 161 | 115 | 67 | 164 | 169 | 80 | 110 |
| The Indian subcontinent | 80 | 92 | 176 | 161 | 121 | 64 | 170 | 169 | 80 | 107 |
| The Indian subcontinent | 83 | 92 | 173 | 170 | 115 | 76 | 170 | 166 | 80 | 98 |
| The Indian subcontinent | 83 | 92 | 179 | 170 | 127 | 67 | 155 | 169 | 92 | 101 |
| The Indian subcontinent | 83 | 92 | 152 | 173 | 124 | 79 | 158 | 166 | 83 | 104 |
| The Indian subcontinent | 83 | 98 | 125 | 176 | 121 | 70 | 161 | 172 | 80 | 101 |
| The Indian subcontinent | 80 | 92 | 179 | 161 | 115 | 64 | 164 | 166 | 80 | 110 |
| The Indian subcontinent | 74 | 95 | 176 | 197 | 118 | 76 | 0 | 154 | 74 | 74 |
| The Indian subcontinent | 80 | 98 | 152 | 161 | 115 | 67 | 167 | 175 | 86 | 104 |
| The Indian subcontinent | 74 | 86 | 176 | 197 | 118 | 67 | 164 | 163 | 74 | 101 |
| The Indian subcontinent | 80 | 92 | 173 | 161 | 0 | 67 | 0 | 172 | 80 | 98 |
| The Indian subcontinent | 80 | 95 | 173 | 170 | 115 | 67 | 161 | 166 | 80 | 98 |
| The Indian subcontinent | 83 | 98 | 158 | 173 | 115 | 67 | 182 | 160 | 71 | 101 |
