# Supplementary material for: Influx of diverse, drug resistant and transmissible Plasmodium falciparum into a malaria-free setting in Qatar
Source: BMC Infect Dis. 2020 Jun 15;20:413. doi: 10.1186/s12879-020-05111-6 (PMC7296620; doi:10.1186/s12879-020-05111-6)
Supplement: Supplementary file 3 — Additional file 3: Table S3. Prevalence of wild-type and mutant alleles of drug resistance genes among imported P. falciparum to Qatar. Values in brackets are percentages. [file 12879_2020_5111_MOESM3_ESM.docx]

**Supplementary Table 3.** Prevalence of wild-type and mutant alleles of drug resistance genes among imported *P. falciparum* to Qatar. Values in brackets are percentages.

|  |  |  | **Wild-type** | | **Mutant** | |  |
| --- | --- | --- | --- | --- | --- | --- | --- |
| **Locus** | **codon change** | **Total samples** | **Africa** | **Asia** | **Africa** | **Asia** | **mixed alleles** |
| **PfMRP1** | H191Y | 70 | 47(67%) | 2(3%) | 15(21%) | 4(6%) | 2(3%) |
|  | K202E | 70 | 53(76%) | 7(10%) | 10(14%) | 0(0%) | 0(0%) |
|  | S437A | 70 | 47(67%) | 2(3%) | 15(21%) | 4(6%) | 2(3%) |
|  | I876V | 70 | 29(41%) | 2(3%) | 27(39%) | 5(7%) | 7(10%) |
|  | L1342M | 70 | 56(80%) | 7(10%) | 7(10%) | 0(0%) | 0(0%) |
|  | F1390I | 70 | 49(70%) | 6(9%) | 13(19%) | 1(1%) | 1(1%) |
|  | K1466R | 70 | 43(61%) | 6(9%) | 15(21%) | 1(1%) | 5(7%) |
|  | D1533V | 70 | 68(97%) | 0(0%) | 2(3%) | 0(0%) | 0(0%) |
| **DHFR** | N51I | 70 | 2(3%) | 5(7%) | 49(70%) | 2(3%) | 12(17%) |
|  | C59R | 70 | 37(53%) | 1(1%) | 18(26%) | 6(9%) | 8(11%) |
|  | S108N | 70 | 3(4%) | 0(0%) | 51(73%) | 7(10%) | 9(13%) |
|  | I164L | 70 | 62(89%) | 7(10%) | 1(1%) | 0(0%) | 0(0%) |
| **Pfmdr1** | N86Y | 70 | 48(69%) | 5(7%) | 21(30%) | 2(3%) | 4(6%) |
|  | Y184F | 70 | 9(13%) | 0(0%) | 47(67%) | 7(10%) | 7(10%) |
|  | F938Y | 70 | 59(84%) | 7(10%) | 3(4%) | 0(0%) | 1(1%) |
|  | G968A | 70 | 53(76%) | 5(7%) | 0(0%) | 2(3%) | 0(0%) |
|  | D1246Y | 70 | 61(87%) | 7(10%) | 1(1%) | 0(0%) | 1(1%) |
| **Pfcrt** | C72S | 70 | 63(90%) | 4(6%) | 0(0%) | 3(4%) | 0(0%) |
|  | K76T | 70 | 62(89%) | 4(6%) | 1(1%) | 3(4%) | 0(0%) |
|  | A220S | 70 | 31(44%) | 1(1%) | 31(44%) | 6(9%) | 1(1%) |
|  | Q271E | 70 | 30(43%) | 5(7%) | 32(46%) | 2(3%) | 1(1%) |
|  | N326D | 70 | 63(90%) | 3(4%) | 0(0%) | 4(6%) | 0(0%) |
|  | N326S | 70 | 43(61%) | 5(7%) | 19(27%) | 2(3%) | 1(1%) |
|  | I356T | 70 | 60(86%) | 6(9%) | 3(4%) | 1(1%) | 0(0%) |
|  | I356L | 70 | 62(89%) | 6(9%) | 2(3%) | 0(0%) | 0(0%) |
|  | R371I | 70 | 31(44%) | 5(7%) | 31(44%) | 2(3%) | 1(1%) |
| **DHPS** | L22F | 70 | 61(87%) | 7(10%) | 2(3%) | 0(0%) | 0(0%) |
|  | I431V | 70 | 61(87%) | 7(10%) | 2(3%) | 0(0%) | 0(0%) |
|  | S436A | 70 | 61(87%) | 7(10%) | 5(7%) | 0(0%) | 2(3%) |
|  | G437A | 70 | 57(81%) | 5(7%) | 4(6%) | 2(3%) | 2(3%) |
|  | K540E | 70 | 16(23%) | 5(7%) | 44(63%) | 2(3%) | 3(4%) |
|  | A581G | 70 | 42(60%) | 6(9%) | 18(26%) | 1(1%) | 3(4%) |
|  | A613S | 70 | 59(84%) | 7(10%) | 2(3%) | 0(0%) | 2(3%) |
| **PFK13** | K108E | 70 | 61(87%) | 7(10%) | 2(3%) | 0(0%) | 0(0%) |
|  | L119L | 70 | 62(89%) | 7(10%) | 1(1%) | 0(0%) | 0(0%) |
|  | H136N | 70 | 62(89%) | 7(10%) | 0(0%) | 0(0%) | 1(1%) |
|  | T149S | 70 | 61(87%) | 7(10%) | 1(1%) | 0(0%) | 1(1%) |
|  | K189T | 70 | 32(46%) | 7(10%) | 25(36%) | 0(0%) | 6(9%) |
|  | K189N | 70 | 61(87%) | 7(10%) | 1(1%) | 0(0%) | 1(1%) |
|  | N217H | 70 | 62(89%) | 7(10%) | 1(1%) | 0(0%) | 0(0%) |
|  | R255K | 70 | 60(86%) | 7(10%) | 2(3%) | 0(0%) | 1(1%) |
|  | I354V | 70 | 62(89%) | 7(10%) | 0(0%) | 0(0%) | 1(1%) |
|  | E433D | 70 | 62(89%) | 7(10%) | 1(1%) | 0(0%) | 0(0%) |
|  | G453A | 70 | 62(89%) | 7(10%) | 1(1%) | 0(0%) | 0(0%) |
